# Data from a survey to determine visitor attitudes and knowledge about the provisioning of wild dolphins at a marine tourism destination

**DOI:** 10.1016/j.dib.2016.11.020

**Published:** 2016-11-12

**Authors:** Greg Simpson, David Newsome, Alicia Day

**Affiliations:** Environmental and Conservation Sciences, Murdoch University, Perth, Western Australia, Australia

## Abstract

In the Austral summer of 2014–2015 we surveyed visitors at the popular marine tourism destination of Koombana Bay, Bunbury, Western Australia to investigate resident and visitor attitudes towards the provisioning of the wild dolphins and their knowledge about the legal, social and environmental repercussions arising from the unregulated provisioning of the dolphins. We report the data collected in our survey along with our preliminary statistical analyses and the survey instrument we utilized to collect the data.

**Specifications Table**

**Table:** 

Subject area	*Environmental Science*
More specific subject area	*Ecotourism Research, Marine Nature Based Tourism, Wildlife Tourism*
Type of data	*Tables and questions from survey instrument*
How data was acquired	*Self-Report Pencil and Paper Questionnaire*
Data format	*Categorized, Analyzed*
Experimental factors	*Data collected in a cross sectional survey research design study*
Experimental features	*Survey opportunistically collected data from visitors engaged in beach orientated summer recreation. We report the survey data for visitor attitudes towards regulated (government licensed) and unregulated provisioning of the wild dolphin population and visitor knowledge about the legal, social and environmental repercussions arising from the unregulated provisioning of the dolphins.*
Data source location	*Koombana Bay, Bunbury, Western Australia (33.3256°S, 115.6396°E)*
Data accessibility	*Data reported in body of article.*

**Value of the data**

• Data and survey instrument questions can be compared with or inform other studies.
• Outcomes of statistical analyses highlight trends in data.
• Provides simple statistical techniques (with exemplars), which may assist other studies.

## 1 Data

We had 116 analyzable questionnaires returned from the 216 we distributed (Females *n*=80 and Males *n*=36). Numerical data for participant responses to categorical, five point Likert scale and ranking questions appear in Table 1, Table 2, Table 3, Table 4, Table 5, Table 6, Table 7. Matched letters in a table indicate statistically significant differences or biases in that data, as confirmed by post hoc testing. We also asked participants three open ended questions that allowed them to explain their attitudes to the provisioning of wild dolphins and their responses appear in Supplementary Table 1–3.

## 2 Experimental design, materials and methods

### 2.1 Rationale for survey site selection

The resident wild population of Indio-Pacific Bottlenose Dolphins (*Tursiops aduncus*) at Koombana Bay in the regional city of Bunbury, Western Australia and the local Dolphin Discovery Centre (DDC) are important drawcards for this marine tourism destination [2], [3], [4]. While visitors can experience the dolphins in regulated encounters coordinated by the DDC, anecdotal evidence suggests that people use private boats to seek out and interact with these wild dolphins on their own terms, which may have a negative impact on the resident dolphin population [1], [5].

### 2.2 Field data collection

We collected our data on two field trips to Koombana Bay during the Austral summer of 2014–2015 by opportunistically sampling visitors using self-report pencil and paper questionnaires in a cross sectional survey research approach. Our survey instrument appears in Supplementary Material: Appendix 1.

### 2.3 Data analysis

Our data analysis primarily utilises chi-squared analysis of categorical data. We use the Marascuilo Procedure for post-hoc testing when statistically significant differences are identified [6]. We apply the Yates Correction in the instances where frequencies of five (5) or less arose [7]. In relation to participant rankings of the likely effectiveness of educational materials, we report mean rankings with the 95% confidence intervals (95%CI) and median values. All analyses utilise data, formulas and functions entered into Microsoft Excel^®^ 2010 spreadsheets.

## Figures and Tables

**Table 1:** Demographic data.

Demographic 1: *Ages of participants*										
Age by Gender	18–25 years	26–35 years		36–45 years		46–55 years		56–64 years		65+ years
Female	12	19		19		17		7		6
Sig. Diff.	No significant bias in age of female participants									
Male	3	7		13		7		3		3
Sig. Diff.	No significant bias in age of male participants									

Demographic 2: *Educational achievement of participants*										
Education by Gender	Year 10	Year 12		Vocational Certificate		Vocational Diploma		Bachelor Degree		Post Graduate
Female	8	13		11		12		18		18
Sig. Diff.	No significant bias in educational achievement of female participants									
Male	6	8		7		3		4		8
Sig. Diff.	No significant bias in educational achievement of male participants									

Demographic 3: *Point of origin for participants*										
Origin by Gender	Bunbury Resident		Perth Resident		Rural Western Australia		Elsewhere in Australia		International Visitor	
Female	20		27		6		11		16	
Sig. Diff.	A		B		A & B					
Male	11		8		3		4		10	
Sig. Diff.	No significant bias in point of origin for male participants									
					Between Gender Difference		Bias in Female Responses		Bias in Male Responses	
				
Age of participants			*χ*^2^ statistics		3.039		0.0234		14.13	
			*p* - values		0.6940		0.9999		0.0148	
				
Education of participants			*χ*^2^ statistics		13.42		0.3111		4.583	
			*p* - values		0.0197		0.9974		0.4688	
				
Origin of participants			*χ*^2^ statistics		2.416		0.0026		7.160	
			*p* - values		0.6957		0.9999		0.1277	

**Table 2:** Attitude of participants towards provisioning the wild dolphin population.

Gender	Support unregulated provisioning	Support regulated provisioning	Do not support any provisioning
Female	3	58	19
Sig. Diff.	A & B	A & C	B & C
Male	3	22	11
Sig. Diff.	D	D & E	E
	Between gender difference	Bias in female responses	Bias in male responses
			
*χ*^2^ statistic	1.778	60.92	15.94
*p* - value	0.4110	<<0.001	0.0003

Regulated Provisioning=Controlled feeding endorsed or licenced by the relevant government agency [1].

Unregulated Provisioning=Anyone feeding wildlife anywhere and anytime contrary to statutory provisions.

**Table 3:** Participant perception of the tourism benefits arising from provisioning the wild dolphins.

Gender	Strongly disagree	Disagree	Not sure	Agree	Strongly agree	Mean response
Female	3	4	5	37	31	4.0 (Agree)
Sig. Diff.	A & B	C & D	E & F	A, C & E	B, D & F	
Male	1	1	1	20	13	4.0 (Agree)
Sig. Diff.	G & H	I & J	K & L	G, I & K	H, J & L	
	Between gender difference		Bias in female responses		Bias in male responses	
			
*χ*^2^ statistic	2.327		71.05		46.13	
*p* - value	0.6759		<<0.001		<0.001	

**Table 4:** Perception of the effectiveness of current penalties for unregulated provisioning wild dolphins.

Gender	Fines decrease unregulated provisioning	Fines do not impact unregulated provisioning	Fines increase unregulated provisioning
Female	36	38	6
Sig. Diff.	B	C & A	B & C
Male	10	26	0
Sig. Diff.	D & E	D, F & A	E & F
	Between gender difference	Bias in female responses	Bias in male responses
			
*χ*^2^ statistic	8.052	53.56	28.69
*p* - value	0.0178	<<0.001	<0.001

**Table 5:** How participants perceive the negative impacts of provisioning wild dolphin populations.

Gender	Strongly disagree	Disagree	Not sure	Agree	Strongly agree		Mean response
Statement 1: *Feeding dolphins can have a negative impact on their health.*							
Female	0	6	18	32	24		3.9 (Agree)
Sig. Diff.	A, B & C	D & E	A	B & D	C & E		
Male	2	5	8	11	10		3.6 (NS-A)
Sig. Diff.	No significant bias in male responses						

Statement 2: *Feeding can cause dolphins to be more attracted to humans.*							
Female	1	2	6	40	31		4.2 (Agree)
Sig. Diff.	A & B	C & D	E & F	A, C & E	B, D & F		
Male	0	6	4	14	12		3.9 (Agree)
Sig. Diff.		G & H		G	H		

Statement 3: *Feeding changes the dolphins’ natural behavior, for example makes them more aggressive if not given food.*							
Female	0	11	32	19	18		4.0 (Agree)
Sig. Diff.	A, B, C & D	A & E	B & E	C	D		
Male	0	5	20	5	6		3.0 (Not Sure)
Sig. Diff.	F	G	F, G, H & I	H	I		

Statement 4: *Feeding dolphins can expose them to unnecessary human associated risks such as entanglement and boat strikes.*							
Female	0	4	9	37	30		4.2 (Agree)
Sig. Diff.	A, B & C	D, E & F	A, D, G & H	B, E & G	C, F & H		
Male	0	6	6	12	12		3.8 (Agree)
Sig. Diff.	I, J, K & L	I, M & N	J, O & P	K, M & O	L, N & P		

Statement 5: *Dolphins can lose their natural ability to hunt on their own if they are fed by humans.*							
Female	0	10	12	30	28	4.0 (Agree)	
Sig. Diff.	A, B C & D	A, E & F	B & G	C, E & G	D & F		
Male	2	8	6	11	9	3.5 (NS-A)	
Sig. Diff.	No significant bias in male responses						
		Between gender differences		Bias in female responses		Bias in male responses	
				
Statement 1	*χ*^2^ statistics	5.124		43.52		8.708	
	*p* - values	0.2748		<0.001		0.0688	
				
Statement 2	*χ*^2^ statistics	9.308		84.47		19.48	
	*p* - values	0.0538		<<0.001		0.001	
				
Statement 3	*χ*^2^ statistics	2.959		35.93		33.22	
	*p* - values	0.5646		<0.001		<0.001	
				
Statement 4	*χ*^2^ statistics	4.730		69.67		15.03	
	*p* - values	0.3161		<<0.001		0.005	
				
Statement 5	*χ*^2^ statistics	4.912		41.52		7.257	
	*p* - values	0.2965		<0.001		0.1229	

**Table 6:** Participant recall of educational materials regarding the provisioning of the wild dolphins.

Gender	Brochure	Newspaper	Signs	Television	Seminars
Female	13	4	11	5	0
Sig. Diff.	A		B		A & B
Male	3	1	4	2	0

Sig. Diff.	No significant bias in male responses				
	Between gender difference		Bias in female responses		Bias in male responses


*χ*^2^ statistic	0.9090		17.34		5.525
*p* - value	0.8233		0.0016		0.2290

**Table 7:** How participants ranked the effectiveness of educational information.

Educational Item	Responses by ranking						Avg. rank±95%CI	Median ranking
	1	2	3	4	5	6		
Female participants (*n*=80 for each item)								
Brochures or flyers.	2	14	25	19	11	9	3.6±0.3	3.0
Signs around beaches, docks and jetties.	49	9	13	6	3	0	1.8±0.2	1.0
Newspaper articles, advertisements, etc.	1	17	20	20	13	10	3.7±0.3	4.0
Television reports, shows, advertisements, etc.	25	25	10	13	6	1	2.4±0.3	2.0
Government supported seminars and talks.	1	2	0	10	11	56	5.4±0.2	6.0
DPAW rangers available for talks.	1	14	11	12	36	6	4.1±0.3	5.0
								
Male participants (*n*=36 for each item)								
Brochures or flyers.	2	9	9	9	5	2	3.3±0.5	3.0
Signs around beaches, docks and jetties.	21	9	5	0	1	0	1.6±0.3	1.0
Newspaper articles, advertisements, etc.	0	7	11	10	7	1	2.6±0.4	3.5
Television reports, shows, advertisements, etc.	10	6	9	8	3	0	2.7±0.4	3.0
Government supported seminars and talks.	0	0	0	2	2	32	5.8±0.2	6.0
DPAW rangers available for talks.	3	7	1	6	18	1	3.9±0.5	5.0

## Footnotes

Transparency document Transparency data associated with this article can be found in the online version at doi:10.1016/j.dib.2016.11.020.
