# Supplementary material for: Data from a survey to determine visitor attitudes and knowledge about the provisioning of wild dolphins at a marine tourism destination
Source: Data Brief. 2016 Nov 12;9:940–5. doi: 10.1016/j.dib.2016.11.020 (PMC5118609; doi:10.1016/j.dib.2016.11.020)
Supplement: Supplementary file 1 — Supplementary material [file mmc1.docx]

None of the authors or Murdoch University have any conflicts of interest in relation to this article.
