# Supplementary material for: Data from a survey to determine visitor attitudes and knowledge about the provisioning of wild dolphins at a marine tourism destination
Source: Data Brief. 2016 Nov 12;9:940–5. doi: 10.1016/j.dib.2016.11.020 (PMC5118609; doi:10.1016/j.dib.2016.11.020)
Supplement: Supplementary file 2 — Supplementary material [file mmc2.docx]

**Appendix 1: Questions from Survey Instrument**

*Demographic data*

- Are you? 🞏 Female 🞏 Male
- Please tick (🗹) your age range:
- 18-25 years 🞏 26-35 years 🞏 36-45 years
- 46-55 years 🞏 56-64 years 🞏 65+ years
- What is the highest level of education you have received?
- Year 10 or equivalent of high school 🞏 Diploma or Advanced Diploma
- Year 12 or equivalent of high school 🞏 Bachelor’s Degree
- VET/TAFE Cert I, II, III, IV or equivalent 🞏 Postgraduate Qualification
- Where is your usual place of residence?
  - Bunbury Resident
  - Perth Metropolitan Area
  - Regional/Rural WA (Please list Postcode)
  - Interstate (Please list Postcode)
  - International (Please list Country)

*Attitudes to provisioning of wild dolphin*

- Bunbury is famous for its resident wild dolphin population in Koombana Bay. Do you believe people should be allowed to feed the dolphins?
  - Yes- Anytime.
  - Yes, but only those permitted as part of a licenced and regulated Department of Parks and Wildlife (DPAW) approved program such as the one that exists at the Bunbury Dolphin Discovery Centre (DDC).
  - No – Dolphins should never be fed by humans.
- Please provide a reason for your answer:

*Knowledge of negative impacts of provisioning of wild dolphin*

- Below are some statements about impacts that could happen as result of humans feeding wild dolphins. Please tick (🗹) the box to indicate how much you agree/disagree with each statement.

| Impacts | 1 Strongly Disagree | 2 Disagree | 3 Not Sure | 4 Agree | 5 Strongly Agree |
| --- | --- | --- | --- | --- | --- |
| *Feeding dolphins can have a negative impact on their health.* |  |  |  |  |  |
| *Feeding can cause dolphins to be more attracted to humans.* |  |  |  |  |  |
| *Feeding changes the dolphins’ natural behaviors (e.g. makes them more aggressive if not given food).* |  |  |  |  |  |
| *Feeding can expose them to unnecessary human associated risks such as entanglement and boat strikes.* |  |  |  |  |  |
| *Dolphins can lose their natural ability to hunt on their own if they are fed by humans.* |  |  |  |  |  |
| *Dolphin feeding benefits tourism.* |  |  |  |  |  |

- Please list any benefits that you see arising from dolphin feeding:

- The last statement in the table above provided the data regarding visitor attitudes to the tourism benefits that may arise from the provisioning of the wild dolphins of Koombana Bay.
- The open ended question provided data for perceived benefits of provisioning of the wild dolphins.

*Recall and rating of educational information*

- Below are some forms of information that inform people of the issues around dolphin feeding.
  1. In the left hand column, please tick (🗹) all that you have seen currently around Bunbury.
  2. In the right column please rank the suitability of each form of information from 1 to 6
     (1 = Most Suitable to 6 = Least Suitable).

| Information | Have you seen any of these around Bunbury? (*Please tick all that apply)* | What forms of these would be suitable to informing people about issues of dolphin feeding?  (*Please rank from 1-6*) |
| --- | --- | --- |
| *Brochures or flyers* |  |  |
| *Signs around beaches, docks and jetties* |  |  |
| *Newspaper articles, advertisements, etc.* |  |  |
| *Television reports, shows, advertisements, etc.* |  |  |
| *Government supported seminars & talks* |  |  |
| *DPAW Rangers available for talks* |  |  |

- The item in the table above about the presence of DPAW Rangers provided the data for visitor contact with Rangers reported with data relating to visitor perceptions of the effectiveness of current fines to deter unregulated provisioning of the Koombana Bay wild dolphin population.

*Perception of the deterrence effect of current penalties*

- Below are some of the penalties for feeding wild dolphins in Western Australia as per Regulation 10 of the *Conservation and Land Management Act 2002* (WA) enforced by DPAW:
  1. $50 on-the-spot fine if caught feeding dolphins outside Department of Parks and Wildlife regulated activities; or
  2. $500 fine if caught feeding dolphins or enticing dolphins with food without authorization or permit if found guilty in court.

Do you believe that these penalties:

- - Decrease the amount of people feed the wild dolphins.
  - Neither increase nor decrease the amount of people feed the wild dolphins.
  - Increase the amount of people feed the wild dolphins.
- Please provide a reason for your answer below:
