# Supplementary material for: Data from a survey to determine visitor attitudes and knowledge about the provisioning of wild dolphins at a marine tourism destination
Source: Data Brief. 2016 Nov 12;9:940–5. doi: 10.1016/j.dib.2016.11.020 (PMC5118609; doi:10.1016/j.dib.2016.11.020)
Supplement: Supplementary file 3 — Supplementary material [file mmc3.docx]

**Supplementary Table 1: Attitudes towards provisioning of wild dolphins**

This table gives participant responses to an open question asking them to rationalise their attitude towards the provisioning of the wild dolphin population of Koombana Bay, Bunbury, Western Australia.

| Participant | Comment |
| --- | --- |
| Male – 26-35 years – International Visitor | Preferably dolphins should not be fed by humans, but I would support controlled feeding if their survival is at risk due to other problems in their habitat caused by humans |
| Male – 26-35 years – Bunbury Resident | I would like the interaction/Good for tourism |
| Male – 46-55 years – Bunbury Resident | Management of anything has costs associated with it some things in life should be free |
| Female – 18-25 years – Bunbury Resident | Everyone should be able to feed the dolphins |
| Female – 36-45 years – Bunbury Resident | They are wild creatures + need to not become dependent on humans to find food |
| Female – 26-35 years – International Visitor | As they are wild dolphins I believe they should not be fed |
| Female – 46-55 years – Perth Resident | There are risks involved for the dolphin (wrong food, uneducated tourist, they seem into net’s or become reliant on the food source and change their natural behaviour |
| Male – 26-35 years – Perth Resident | They don’t need assistance, they can get food themselves |
| Male – 26-35 years – International Visitor | Being able to interact with the dolphins probably encourages people to visit the discovery centre, this provides an opportunity to educate them |
| Female – 26-35 years – International Visitor | They are wild animals, hence they are & should be able to feed themselves. If they are fed by humans I believe they could easily become dependent on that, and secondly start associating humans with food-not healthy behaviour. |
| Female – 36-45 years – Regional/Rural WA | They are wild animals. Feeding takes away natural instinct |
| Male – 65+ years – International Visitor | Dolphins are wild animals. Feeding them alters their natural behaviour and induces dependency and can be detrimental to long term survival of the species. |
| Female – 65+ years – Australia outside WA | It seems that, by feeding, it encourages the dolphins to visit, thereby allowing humans to see them up close |
| Female – 65+ years – Perth Resident | Safety of dolphins |
| Female – 65+ years – Perth Resident | Not to harm the dolphins doing so |
| Female – 56-64 years – International Visitor | People cannot be trusted to feed correctly; increased interaction from food expectations creates hazards + accidents |
| Female – 18-24 years – International Visitor | Because: - development of tourism   -interaction with dolphin and human But: not every time just during a part of the days or night |
| Male – 18-25 –  Regional/Rural WA | Develop tourism in Bunbury |
| Female – 18-25 –  Australia outside WA | It is a great way to encourage people to learn more about dolphins |
| Male – 26-35 years – International Visitor | They may become too “tame” |
| Female – 36-45 –  Australia outside WA | If people can interact with natural environment, it can lead to necessary behaviour changes at home re: environmental protection |
| Male – 36-45 years – Bunbury Resident | Tourism Research by DDC important |
| Female – 46-55 years – Bunbury Resident | Needs to be moderated |
| Female – 36-45 years – Bunbury Resident | Animals becoming too domesticated |
| Female – 26-35 years – Bunbury Resident | Dolphin health |
| Female – 18-25 years – Bunbury Resident | Wild animals should be left as a wild animal  Might hurt dolphins – wrong food |
| Female – 46-55 years – Bunbury Resident | Well, if we allow anyone to feed dolphins, they could be fed potentially harmful food |
| Male – 46-55 years – Bunbury Resident | Not a natural thing to do – dolphins become to [sic] friendly and people take advantage of them. Become reliant – scavengers not hunters |
| Male – 65+ years – Regional/Rural WA | Leave wildlife to own sources |
| Female – 56-64 years – Bunbury Resident | No harm in feeding  DDC costs money - affordability |
| Male – 46-55 years – Australia outside WA | Feeding in a controlled environment is ok |
| Male – 65+ years – Australia outside WA | Need to keep the dolphins safe from poor feeding  Good for conservation |
| Male – 36-45 years – Bunbury Resident | Feeding outside the centre is unnecessary  Dolphins are friendly otherwise |
| Male – 65+ years – Bunbury Resident | Free feeding is unsafe for the dolphins |
| Female – 36-45 years – Perth Resident | There’s got to be some regulation |
| Female – 65+ years – Bunbury Resident | They shouldn’t be fed all the time – diseases, get into bad habits ie become dependent or aggressive |
| Male – 36-45 years – Bunbury Resident | Makes people aware – good educational tool |
| Female – 26-35 years – Regional/Rural WA | These programs offer a chance to educate the general public about our beautiful wild animals |
| Female – 36-45 years – Bunbury Resident | I think that the feeding of dolphins in a regulated manner helps with the education of the public |
| Female – 36-45 years – Bunbury Resident | The dolphins need to know how to find food for themselves, and be able to teach their young how to find food |
| Male – 46-55 years – Rural WA | People are ‘wallies’ – can’t be trusted to do the right thing by the dolphins |
| Female – 26-35 years – Bunbury Resident | Because hopefully this has been successfully researched that it provides the ability for people to feed dolphins without them relying on people for food |
| Female – 56-64 years – Perth Resident | So don’t overfeed them |
| Female – 36-45 years – Australia outside WA | There is a chance dolphins might become partly dependent on human feeding and should be avoided. However, there might be circumstances in the future such as temporarily feeding e.g. if water is polluted until levels return to normal, or lack of fish in the bay. Important would be to feed living fish to maintain hunting abilities. Other than that I cannot think of any other reason at this stage. It is wonderful that dolphins are willing to come in without wanting/needing to be fed. |
| Female –18-25 years – International Visitor | Actually I am against human feeding of dolphins but when I am honest I enjoy watching them as well |
| Male –18-25 years – International Visitor | Because they are WILD |
| Female – 26-35 years – Perth Resident | Safest option for the dolphins |
| Female – 26-35 years – Bunbury Resident | None. The DDC must exist for a reason. |
| Female – 26-35 years – Australia outside WA | Dolphins choose to feed, they are not force-fed |
| Female – 26-35 years – Perth Resident | Because they are wild animals and should be able to fend for themselves and not rely on human intervention |
| Female – 18-25 years – International Visitor | If it’s been found to be safe for the dolphins then it is good for everyone |
| Female – 46-55 years – Australia outside WA | As long as it’s by people who know what they are doing |
| Male – 36-45 years – International Visitor | The experts know what they’re doing |
| Male – 56-64 years – Perth Resident | People need to interact with wildlife in a restricted manner |
| Female – 56-64 years – Perth Resident | They should be left to hunt and feed naturally |
| Female – 46-55 years – Perth Resident | Feeding outside of controlled conditions interferes with natural habits |
| Female – 46-55 years – Perth Resident | No problem with feeding with small amount-small scraps won’t hurt them |
| Male – 56-64 years – Bunbury Resident | You don’t know what other people feed them i.e. rubbish |
| Male – 46-55 years – Bunbury Resident | To monitor the type of food stuff fed to them |
| Female – 26-35 years – Perth Resident | People are going to do it anyway, so if it’s in a controlled environment then it’s good and then they can be educated |
| Female – 36-45 years – International Visitor | It is important that wildlife stays wild and don’t get sick of wrong food. But to keep interest up and respect for the wild animals it is important to be able to see them up close in their natural environment and her the Discovery Centre plays an important role so it is good they can feed the dolphins as a treat |
| Female – 18-25 years – Perth Resident | The food can be harmful to dolphins. Damage to natural behaviours.  But good for tourism |
| Female – 26-35 years – Perth Resident | In order to continue to survive |
| Female – 26-35 years – Perth Resident | Controlled feeding will allow the dolphins to remain wild |
| Female – 36-45 years – Perth Resident | Enabling increased interaction and/or encouraging more consistent visits by the dolphins I’d envisage would have significant benefits for the centre and tourism. With presumably limited funding, the centre would require as much public support as possible |
| Female – 26-35 years – Bunbury Resident | If dolphins are fed by humans all the time it will force habits and they will rely on this method of feeding become incapable of hunting in the wild etc. thus making them vulnerable |
| Female – 26-35 years – Bunbury Resident | Don’t want them dependant on us for food |
| Female – 26-35 years – Perth Resident | They should be left alone they are wild dolphins |
| Female – 56-64 years – Regional/Rural WA | Dolphins know the DDC is a safe place |
| Male – 36-45 years – Australia outside WA | I would also add that feeding may be allowed to help and injured or sick animal if this was found to be helpful |
| Female – 18-25 years – International Visitor | - They’re wild animals and have their own environment - Trust in humans - Come close to fishers |
| Female – 46-55 years – International Visitor | Keep them as wild you can |
| Female – 26-35 years – International Visitor | They can catch their food by his own |
| Female – 36-45 years – International Visitor | Concern for dolphin health and conservation. Not healthy for general public feeding to feed them --> wrong food |
| Male – 36-45 years – Australia outside WA | There is no sustainability issue that I am aware of. While it may not be good for the dolphin’s diet, they’re smart animals. Up to them. |
| Female – 46-55 years – Regional/Rural WA | Not impacting dolphin’s natural behaviour |
| Female – 36-45 years – Bunbury Resident | Nice to have the close encounter but concerned about dolphin welfare |
| Female – 65+ years – International Visitor | Dolphins are dolphins and not seagulls. However alternative 2 allows for research, it’s done properly and gives us an experience on the dolphins terms. |

| Male – 65+ years – International Visitor | Dolphins and other wild animals shouldn’t get accustomed to human feeding because it distracts their natural feeding behaviour and most human manufactured good contain nutrients and additives that would not occur in their usual diet and might have adverse effects on their physiology |
| --- | --- |
| Female – 65+ years – International Visitor | I think the dolphin that live here are wild animals and should be treated as such |
| Female – 36-45 years – Perth Resident | If volunteers at the centre fed one fish per dolphin it would keep dolphins and humans interactive – without dolphins dependency on human feeding, and may prevent external dolphin feeding activities elsewhere. However I believe dolphins are attracted to humans for their company and socialization. |
| Male – 36-45 years – Australia outside WA | Tourism |
| Female – 46-55 years – Perth Resident | That way anyone can enjoy them or else you get idiots throwing fishing lines in them, by doing it with permission it keeps them safe |
| Female – 46-55 years – Bunbury Resident | Encourage any wild animal to feed they’ll lose their ability on their own |
| Male – 36-45 years – Perth Resident | The wildlife people are trained and have the dolphins best interest foremost more so than the average joe |
| Male – 36-45 years – International Visitor | Generally I think don’t feed dolphins or any wild animal ever, however having seen the practice here I would suggest you have it right and the benefit for both the dolphin and humans to see/interact in this way can only be positive |
| Female – 46-55 years – Australia outside WA | As they are a wild animal it would upset the balance. They need to hunt to find their own food, leave well alone! |
| Male – 36-45 years – International Visitor | I think wild animals should be left to find their own food, however I think it is ok if it licensed and used only to research the local dolphin population, habits, health etc. |

**Supplementary Table 2: Perceived benefits of provisioning wild dolphin population**

Participant responses to an open question that asked them to list any benefits they saw arising from provisioning the wild dolphin population of Koombana Bay, Bunbury, Western Australia.

| Participant | Comment |
| --- | --- |
| Male – 26-35 years – International Visitor | Younger generations can learn to love and respect dolphin life in a controlled practical environment, which would assist their protection in next generations |
| Male – 26-35 years – Bunbury Resident | Attracting more dolphins to the discovery centre. Good for tourism. Only under controlled circumstances |
| Male – 46-55 years – Bunbury Resident | Human contact with nature |
| Female – 36-45 years – Perth Resident | Creates assurance that tourists will see dolphins |
| Female – 18-25 years – Bunbury Resident | If its [sic] just feeding when people are there to watch what they are given, it would be good and bring in more tourism if people could help to feed them fish etc. |
| Female – 26-35 years – International Visitor | If dolphins are being fed legally for conservation purposes |
| Female – 46-55 years – Perth Resident | Interaction with humans are of benefit for humans and therefore it enhances our interest to conserve this part of wildlife |
| Male – 26-35 years – International Visitor | Only controlled feeding at centres in order to attract tourists and educate them about dolphins |
| Female – 26-35 years – International Visitor | They’d be guaranteed food, I guess… |
| Male – 65+ years – International Visitor | None to the dolphins – the benefit is to tourists who want to be guaranteed dolphin sightings |
| Female – 26-35 years – Perth Resident | Tourism |
| Female – 65+ years – Australia outside WA | Behavioural patterns can be observed |
| Female – 65+ years – Perth Resident | As above |
| Male – 36-45 years – Perth Resident | No- I believe that not feeding dolphins is the way forward as they very good natural hunters and shouldn’t lose this ability |
| Female – 18-24 years – International Visitor | -more tourism  -may be help to study dolphins  -may be help to count dolphin |
| Male – 18-25 –  Regional/Rural WA | Attractive more tourism |
| Female – 36-45 –  Australia outside WA | Younger generations can learn to love and respect dolphin life in a controlled practical environment, which would assist their protection in next generations |
| Male – 36-45 years – Bunbury Resident | Benefit to economy via tourism Research |
| Female – 46-55 years – Bunbury Resident | Tourism  Good for disabilities |
| Female – 26-35 years – Bunbury Resident | One on one time with dolphins – special interaction |
| Female – 18-25 years – Bunbury Resident | The DDC can monitor the dolphin’s health |
| Female – 46-55 years – Bunbury Resident | Dolphins might come around more often so we can see them. Will attract more tourists |
| Male – 36-45 years – Bunbury Resident | Not many |
| Male – 46-55 years – Bunbury Resident | It gives humans an opportunity to interact but not good for dolphins |
| Female – 56-64 years – Bunbury Resident | Tourism |

| Male – 46-55 years – Australia outside WA | Tourism |
| --- | --- |
| Male – 65+ years – Australia outside WA | Education |
| Male – 36-45 years – Bunbury Resident | Tourism |
| Male – 65+ years – Bunbury Resident | Tourism/economic  Improved understanding by the general public |
| Female – 36-45 years – Perth Resident | No feed = no interaction  Good for conservation – study and research |
| Female – 65+ years – Bunbury Resident | Tourism – but it only benefits people, not dolphins |
| Male – 36-45 years – Bunbury Resident | It only benefits people – not dolphins. It’s only our perception that thinks they benefit |
| Female – 26-35 years – Regional/Rural WA | Places like the Dolphin Centre attract visitors. It is a great opportunity to educate people about the animals and their habitats |
| Female – 36-45 years – Bunbury Resident | See question 9 [Table 3 above] |
| Female – 36-45 years – Bunbury Resident | May support the dolphins when fish stocks are low |
| Male – 46-55 years – Regional/Rural WA | None  Good experience for humans |
| Female – 26-35 years – Bunbury Resident | Closer interaction with the dolphins – hopefully they will always show up to be fed |
| Female – 56-64 years – Perth Resident | Good for humans |
| Female – 36-45 years – Australia outside WA | More dollars/tourism for the operators however greed cannot benefit anyone really |
| Female –18-25 years – International Visitor | It is nice for tourists to see wild dolphins in their natural environment. Maybe the feeding by humans could help weaker dolphins which couldn’t survive on their own. |
| Male –18-25 years – International Visitor | Tourism |
| Female – 26-35 years – Perth Resident | Tourism or reintegration into the wild from injury |
| Female – 26-35 years – Bunbury Resident | People like seeing them up close |
| Female – 26-35 years – Australia outside WA | People and nature co-exist together. That is the reality of our world. They are exposed to each other. |
| Female – 18-25 years – International Visitor | Regulated safe. Unregulated unsafe for the dolphins |
| Female – 46-55 years – Australia outside WA | If done by experts in a controlled environment then it’s good for dolphin conservation because they can on health, numbers etc. like at the DDC (get to know dolphins) |
| Male – 36-45 years – International Visitor | Tourism |
| Male – 56-64 years – Perth Resident | Research in feeding habits. Good for tourist |
| Female – 56-64 years – Perth Resident | Could help in some research. Attack tourism to a local area-employ people?!? |
| Female – 46-55 years – Perth Resident | Tourism purposes. If the dolphins are injured or need rehabilitation the feeding helps take care of them |
| Male – 56-64 years – Bunbury Resident | Not really-I would prefer they fed on their own so they don’t lose their hunting ability |
| Male – 46-55 years – Bunbury Resident | None that I can think of |
| Female – 26-35 years – Perth Resident | Education and a “lifetime” experience especially for young people like little kids |
| Female – 36-45 years – International Visitor | See number 9 [Table 3 above] |
| Female – 18-25 years – Perth Resident | Tourism-economy  But not beneficial to dolphins |
| Female – 26-35 years – Perth Resident | Tourism, educational experiences for children. Increased understanding of wildlife |
| Female – 36-45 years – Perth Resident | Regulated and controlled feeding is conducted to minimise negative impacts but there are significant gains for tourism and engagement with wild animals |
| Female – 26-35 years – Bunbury Resident | Money making with regards to tourism |
| Female – 26-35 years – Bunbury Resident | Better understanding of dolphins so hopefully people are more careful with throwing rubbish and alike away |
| Female – 26-35 years – Perth Resident | Tourism ie Monkey Mia is packed every day. Good for conservation for sick dolphins. Good for education for young kids |
| Female – 56-64 years – Regional/Rural WA | Regulated feeding benefits tourism |
| Male – 36-45 years – Australia outside WA | Selective feeding can help in the study of dolphins which will lead to benefits for the dolphins in the long term |
| Female – 26-35 years – International Visitor | Can’t see any benefits |
| Female – 36-45 years – International Visitor | General awareness of ecology and environmental conservation by humans |
| Male – 36-45 years – Australia outside WA | Humans feed lots of animals. Even simple companionship [sic] can be a benefit |
| Female – 36-45 years – Australia outside WA | Good for conservation if injured or sick-sometimes we have to intervene if sick |
| Female – 46-55 years – Rural WA | Tourism. It’s really good for people with autism-learn to be kind to animals |
| Female – 36-45 years – Bunbury Resident | Tight regulated small amount of food allows us to have a safe encounter |
| Female – 46-55 years – International Visitor | Educational benefits, and just benefits for the people to get closer to the dolphins |
| Male – 65+ years – International Visitor | Like the centre is doing it, according to scientific data (I presume), and to attract attention in a non-disruptive way, I think it may have a positive impact on awareness |
| Female – 65+ years – International Visitor | The only benefit I can see is tourism and money, but I don’t think that that should be count in the discussion. Wild animals are not our little pot of gold… |
| Female – 36-45 years – Perth Resident | Increased in interactive and tourism for humans.   - Centre staff maybe able to get closer to the dolphins to monitor their health - Over hand feeding dolphins through I believe will have a detrimental effect on the dolphins feeding in the wild. However we all like “snack time” |
| Male – 36-45 years – Australia outside WA | Tourism/Business side. Feeding a little benefits tourism industry-dolphins won’t come back if they don’t get anything. |
| Female – 46-55 years – Perth Resident | Pleasing people. The dolphins enjoy it, they enjoy the interaction |
| Female – 46-55 years – Bunbury Resident | Tourism |
| Male – 36-45 years – Perth Resident | Tourism. Kids enjoy it and are more likely to have compassion for them if they can see them up close |
| Female – 46-55 years – Regional/Rural WA | Better understanding of dolphin behaviour |
| Male – 36-45 years – International Visitor | - Human-dolphin interaction - Opportunity for children/ adults to be close to dolphins |
| Female – 46-55 years – Australia outside WA | It would only benefit the enjoyment of the humans |
| Male – 26-35 years – Perth Resident | Nil benefits for the dolphins |
| Male – 36-45 years – International Visitor | Only when licensed the ability to research health, population etc |

**Supplementary Table 3: Effectiveness of current penalties to deter unregulated provisioning**

Participant responses to an open question that asked them fines provisioning the wild dolphin population of Koombana Bay, Bunbury, Western Australia.

| Participant | Comment |
| --- | --- |
| Male – 26-35 years – International Visitor | Fines aren’t stringent enough |
| Male – 26-35 years – Bunbury Resident | I think the fine is sufficient to deter feeding |
| Male – 46-55 years – Bunbury Resident | Some things in life should be free |
| Male – 36-45 years – Bunbury Resident | No idea there was a fine involved. |
| Female – 36-45 years – Bunbury Resident | No idea there was a fine involved. |
| Male – 46-55 years – Bunbury Resident | Until I filled out this form, I was unaware of the penalties. I have seen unauthorised feeding a lot. |
| Female – 26-35 years – International Visitor | Fines are usually deterrents |
| Female – 46-55 years – Perth Resident | Because there is no awareness about this fines |
| Male – 26-35 years – International Visitor | Encourage people not to feed dolphins |
| Female – 26-35 years – International Visitor | The penalties remind me that dolphin feeding is harmful and therefore made illegal |
| Female – 36-45 years – Regional/Rural WA | Fines aren’t stringent enough |
| Female – 26-35 years – Perth Resident | Minimally – not really policed |
| Female – 65+ years – Australia outside WA | Fines can be a deterrent |
| Female – 65+ years – Perth Resident | Fine should be higher |
| Female – 65+ years – Perth Resident | Fine should be higher |
| Female – 56-64 years – International Visitor | Penalties should be higher than a viewing trip |
| Female – 18-24 years – International Visitor | It’s not expensive so may be $1000 better… |
| Male – 18-25years –  Regional/Rural WA | It’s not expensive |
| Female – 18-25 –  Australia outside WA | I believe these measures would only deter a small group of people, therefore decreasing the amount of people feeding dolphins but not to a large extent |
| Male – 26-35 years – International Visitor | Monetary punishments |
| Female – 36-45 –  Australia outside WA | People don’t like to pay fines! |
| Female – 26-55years –  Regional/Rural WA | It would probably be rare to get caught. Those wanting to do the right thing always will anyhow a $50 fine would not stop silly people, too light, small risk of getting caught |
| Male – 36-45 years – Bunbury Resident | Didn’t know there were fines |
| Female – 46-55 years – Bunbury Resident | Fines not heavy enough  Not sure many people feed dolphins |
| Female – 36-45 years – Bunbury Resident | More boats out and the dolphins around  Lack of policing |
| Female – 26-35 years – Bunbury Resident | People will do it anyway |
| Female – 18-25 years – Bunbury Resident | No one wants to get fined |
| Female – 46-55 years – Bunbury Resident | If people realise there’s a fine, they’ll stop feeding them so they don’t get charged |
| Male – 46-55 years – Bunbury Resident | Wasn’t aware of the legislation or fines – no signs |
| Male – 65+ years – Regional/Rural WA | Not enough policing/enforcement |
| Male – 65+ years – Australia outside WA | People will ignore the fines  Penalties aren’t harsh enough |
| Male – 36-45 years – Bunbury Resident | Haven’t heard about the penalties |
| Male – 65+ years – Bunbury Resident | Penalty not harsh enough |
| Female – 36-45 years – Perth Resident | It’s a deterrent but people need to know the fines exist |
| Female – 65+ years – Bunbury Resident | $50 doesn’t seem harsh enough |
| Male – 36-45 years – Bunbury Resident | It’s too hard to regulate – people will do it anyway (we need correct education) |
| Female – 26-35 years – Regional/Rural WA | Generally the kinds of people who ignore the advice and guidelines are also probably going to ignore the fine risks |
| Female – 36-45 years – Bunbury Resident | If the knowledge i.e. fines was known by the general public I think it would decrease |
| Female – 36-45 years – Bunbury Resident | Most people wouldn’t know what the penalty is, I didn’t know |
| Male – 46-55 years – Regional/Rural WA | Didn’t know there were penalties |
| Female – 26-35 years – Bunbury Resident | I don’t know how many people know about these laws |
| Female – 56-64 years – Perth Resident | Protect dolphins |
| Female – 36-45 years – Australia outside WA | I have no insight on this but am wondering how often these regulations are patrolled/ checked |
| Female –18-25 years – International Visitor | If people feed the dolphins they think they will get caught |
| Female – 26-35 years – Perth Resident | No communication or known enforcement of these fines |
| Female – 26-35 years – Bunbury Resident | Don’t know about regs [sic – regulations]. Offenders are tourists. |
| Female – 36-45 years – Perth Resident | People’s tendency is to avoid playing penalties |
| Female – 26-35 years – Australia outside WA | People do what they want regardless of the consequences, look at crime in our society |
| Female – 26-35 years – Perth Resident | Monetary fines generally discourage people |
| Female – 36-45 years – Perth Resident | Fines normally deter but the problem is catching people in the act |
| Female – 46-55 years – Australia outside WA | People always try to push the limits and have no regard for authorities |
| Female – 18-25 years – International Visitor | Hopefully the penalties are effective |
| Female – 46-55 years – Australia outside WA | People don’t know about fines. Fines aren’t harsh enough (people don’t care about $50) |
| Male – 36-45 years – International Visitor | Penalties are working- of course they do |
| Female – 56-64 years – Perth Resident | If the public are aware of the fines system it could deter them |
| Female – 46-55 years – Perth Resident | Didn’t know penalties applied and not sure how well known they are |
| Female – 46-55 years – Perth Resident | People don’t want to be fined |
| Male – 56-64 years – Bunbury Resident | People will feed them anyway |
| Male – 46-55 years – Bunbury Resident | I believe ppl [sic – abbreviation for people] will feed them regardless as we cannot help ourselves |
| Female – 26-35 years – Perth Resident | People will do it anyway and there’s not enough education out there-people don’t know about fines |
| Female – 36-45 years – International Visitor | It is a very monetary world and people don’t want to pay penalties. On the other hand the world is very individualistic and people tend to what suits them and therefore knowledge and respect are important. |
| Female – 18-25 years – Perth Resident | No one wants to get fined Only makes a small difference – no policing |
| Male – 46-55 years – Perth Resident | Not strong enough fines |
| Female – 26-35 years – Perth Resident | To discourage people. $500 is pretty cheap to feed a dolphin. |
| Female – 26-35 years – Perth Resident | Penalties should deter, but only if people are aware of the consequences |
| Female – 36-45 years – Perth Resident | However, only if people are educated and aware of the penalties. We only learnt about the issue when participating in dolphin swim and there were nearby boats with people jumping in water |
| Female – 26-35 years – Bunbury Resident | If you don’t get caught there is no fine! |
| Female – 26-35 years – Bunbury Resident | I think if people want to do it then will regardless of penalty |
| Female – 26-35 years – Perth Resident | I didn’t know there was a fine, but penalties aren’t harsh enough |
| Female – 56-64 years – Regional/Rural WA | Penalty not harsh enough |
| Male – 36-45 years – Australia outside WA | Without publicising that dolphin feeding is illegal, people will feed/or not for moral reasons rather than because of the fine |
| Female – 18-25 years – International Visitor | If you wanna [sic – colloquialism of ‘want to’] feed them you’ll do it anyways [sic] and just try not to get caught |
| Female – 18-25 years – International Visitor | - It’s hard to control - Makes it for some people more attractive, others are scared |
| Female – 46-55 years – International Visitor | Not harsh enough penalties and not policed enough-hard to catch/prosecute |
| Female – 26-35 years – International Visitor | People don’t like to pay fines! |
| Female – 36-45 years – International Visitor | It’s a dolphin. I didn’t even know there was a law |
| Female – 36-45 years – Australia outside WA | Not harsh enough $50 is nothing. I’ll pay that to feed a dolphin |
| Female – 46-55 years – Regional/Rural WA | Weren’t aware of the penalties |
| Female – 36-45 years – Bunbury Resident | Didn’t know there were fines |
| Female – 46-55 years – International Visitor | If people know about those fines, they will try to avoid being caught and [unintelligible]. It’s all about control and amount of rangers. |
| Male – 65+ years – International Visitor | If there is a sufficient “chance to get caught” it will act as deterrant [sic], but like in most cases, just the rule being in place won’t achieve much |
| Female – 65+ years – International Visitor | When it is clear that feeding dolphin is illegal less people will be inclined to do so |
| Male – 36-45 years – Australia outside WA | Wasn’t aware of fines – tourists especially don’t know |
| Female – 46-55 years – Perth Resident | People that want to feed the dolphins they will – you may scare the odd tourist |
| Female – 46-55 years – Bunbury Resident | People aren’t aware of the fines – I assumed there was but didn’t really know |
| Male – 36-45 years – Perth Resident | People are not education on the affect it could have on them |
| Female – 46-55 years – Regional/Rural WA | Not enforced enough through lack of rangers and little understanding by general public |
| Male – 36-45 years – International Visitor | Most people won’t make the effort to do so. However if you were that way inclined this deterrent would probably not stop you |
| Female – 46-55 years – Australia outside WA | A large fine would surely deter people from feeding the dolphins |
| Male – 26-35 years – Perth Resident | Penalties aren’t harsh enough and there is no common knowledge/education about penalties |
